# Epiphytic Patterns Impacting Metabolite Diversity of *Drynaria roosii* Rhizomes Based on Widely Targeted Metabolomics

**DOI:** 10.3390/metabo14080409

**Published:** 2024-07-26

**Authors:** Nana Chang, Xianping Yang, Xiaoqing Wang, Chao Chen, Chu Wang, Yang Xu, Hengyu Huang, Ye Wang

**Affiliations:** 1 Jiangxi Province Key Laboratory of Sustainable Utilization of Traditional Chinese Medicine Resources, Institute of Traditional Chinese Medicine Health Industry, China Academy of Chinese Medical Sciences, Nanchang 330115, China; 2 Jiangxi Institute of Traditional Chinese Medicine Health Industry, Nanchang 330115, China; 3 Dexing Research and Training Center, Dexing Academy of Traditional Chinese Medicine, Dexing 334213, China; 4 Jiangxi Provincial Institute of Traditional Chinese Medicine, Nanchang 330046, China; 5 College of Traditional Chinese Medicine, Yunnan University of Chinese Medicine, Kunming 650500, China

**Keywords:** *Drynaria roosii*, rhizomes, epiphytic patterns, metabolomics, medicinal herb

## Abstract

*Drynaria roosii* Nakaike, a fern widely distributed in China and some countries in Southeast Asia, is a commonly used herbal medicine in tonic diets and Chinese patented medicine. The metabolites of its dried rhizomes are easily affected by the epiphytic pattern, whether on rock tunnels (RTs) or tree trunks (TTs). The current research focused on rhizomes from these two patterns, RTs and TTs (further divided into subclasses TA, TB, TC, and TD, based on trunk differences) and conducted a widely targeted metabolomics analysis. A total of 1435 components were identified across 13 categories, with flavonoids, amino acids, and their derivative, lipids, identified as the main components. They accounted for 19.96%, 12.07%, and 12.14% of all metabolites, respectively. The top five flavonoids in TB were eriodicty-ol-7-*O*-(6″-acetyl)glucoside, quercetin-3-*O*-sophoroside (baimaside), dihydrochar-cone-4′-*O*-glucoside, morin, and hesperetin-7-*O*-glucoside, with relative contents 76.10, 24.20, 17.02, 15.84, and 14.64 times higher than in RTs. Principal component analysis revealed that samples with different epiphytic patterns clustered into five groups. The RT patterns revealed unique metabolites that were not detected in the other four epiphytic species (TA, TB, TC, and TD), including 16 authenticated metabolites: 1 alkaloid, 1 amino acid derivative, 7 flavonoids, 2 lignans, 1 lipid, 1 alcohol, 1 aldehyde, and 2 phenolic acids. These differences in epiphytic patterns considerably affected the accumulation of both primary and secondary metabolites. The comparison of diversity between RTs and TTs can guide the selection of a cultivation substance and the grading of collective rhizomes in the wild. This comprehensive analysis of *D. roosii* rhizome metabolites also offers fundamental insights for identifying active components and understanding the mechanisms underlying their potential pharmacological activities.

## 1. Introduction

*Drynaria roosii* is a fern belonging to the Drynariaceae family, *Drynaria* genus, which possesses medicinal and ornamental value attributed to extensive rhizomes and distinctive fronds. The dried rhizomes serve as the primary source of Drynariae Rhizoma, a Chinese herb used for various purposes such as treating kidney deficiency, muscles tears, loose teeth when taken orally, and white smallpox and alopecia in the form of an externally applied agent on topical areas [1]. The efficiency of Drynariae Rhizoma has been demonstrated in East Asia for promoting the proliferation and differentiation of osteogenesis as well as alleviating house dust mite antigen-induced atopic dermatitis [2,3]. Flavonoids are key components that promote the proliferation and differentiation of MC3T3-E1 cells and prevent acute renal failure [4,5]. Currently, Drynariae Rhizoma is included in approximately 183 Chinese patented medicines, with an estimated annual demand of up to 6,500,000 kg in China based on our investigation. These patented medicines, including Drynariae Rhizoma, have contributed to curing bone fractures in the elderly. The demand for rhizomes has increased owing to an aging population in certain countries.

As a perennial epiphyte, *D. roosii* mainly grows on old tree trunks, limestone, and even buildings. Epiphytic growth patterns considerably influence secondary metabolites, thereby affecting the plant’s nutrient and medicinal values. Different epiphytic patterns generate a differential impact on chemical components and pharmacological functions because of differing ecological factors and growth environments, such as sunlight exposure and soil properties [6,7]. Similar studies on epiphytic plants, such as *Dendrobium nobile*, revealed that epiphytic patterns (Danxia stone, crushed stone, sawdust, and stump) induced a series of metabolic changes at the metabolome level and the synthesis of amino acids and terpenoids was increased in Danxia stone more than in the other cultivation patterns [8]. Notably, perennial epiphytes exhibit specific traits, where the same botanical part displays varying chemical compositions depending on its growth age. For example, the alkaloid content of *D. nobile* decreases with prolonged cultivation age, whereas glucosides exhibit the opposite trend [9,10]. Long-term cultivation in a wild-growth environment can promote the accumulation and enrichment of secondary metabolites, such as flavonoids and phenolic acids in *D. nobile*, enhancing its clinical efficiency [11]. Furthermore, variations in chemical composition with growth years lead to differences in protective effects against liver injury [12]. However, the metabolites of *D. roosii*, a perennial epiphyte with growth patterns similar to those of *D. nobile*, require further investigation for scientific usage.

While some studies have focused on gene expression and content variation in specific compounds, such as naringin/neoeriocitrin [1,13,14,15], limited research has been conducted to elucidate the metabolite variations caused by differing epiphytic patterns. Epiphytic fern assemblages in wet, warm climates at low latitudes and elevations are strongly influenced by climatic factors [16]. Much attention has been directed to revealing the effects of epiphytic patterns. Incorporating an additional epiphytic species allows for a broader exploration of metabolite variations due to species diversity, growth age, and botanical parts.

Metabolomics, an inclusive protocol for characterizing the quality and constituents of medicinal plants, employs metabolite profiles in conjunction with multivariate statistical analyses [17,18]. This method compensates for the deficiency that some microconstituents fail to be detected by traditional technologies. Widely targeted metabolomics (WTM) combines the advantages of both targeted and untargeted metabolomics, offering qualitative and quantitative accuracy, wide coverage, and high throughput [18,19,20]. This technology has been widely used for quality assessment and control in traditional Chinese medicine. Differential metabolites detected by WTM indicate better quality in wild samples than in cultivated *Rehmannia glutinosa*, with 75.9% of differential metabolites being upregulated and 30 unique components in the wild group [18]. Quercetin, eriodictyol, and many flavone C-glycosides are significantly enhanced after flooding stress based on WTM in *Chrysanthemum morifolium* at two developmental stages [21].

In this study, we extensively examined metabolite variations in *D. roosii* rhizomes collected from various epiphytic patterns using ultra-performance liquid chromatography–electrospray ionization–mass spectrometry (UPLC-ESI-MS/MS) and widely targeted metabolomics analysis. We also investigated the differences in metabolites caused by epiphytic tree species, providing scientific insights into the rational and efficient use of *D. roosii* rhizomes. This integrated evaluation provides valuable references on metabolites for the further development and utilization of fern resources, as well as for sustainable harvest and processing in the regions to which *D. roosii* is native.

## 2. Materials and Methods

### 2.1. Rhizome Sample Collection and Chemical Reagents

Wild rhizomes were collected from Huaqiao Country in Dexing City, Jiangxi Province, China (28.95 N, 117.76 E). The collection site is located in a subtropical humid monsoon zone with a climate featuring abundant rainfall, sufficient sunlight, and distinctive seasons. The fern was authenticated as *Drynaria roosii* Nakaike by Dr. Dan Xie from the South China Botanical Garden, Chinese Academy of Sciences, in Guangdong, China. The rhizomes were grown in two epiphytic patterns: rock tunnels (RTs) and tree trunks (TTs). The tree trunk pattern contained four overgrowth species, including *Clerodendrum mandarinorum* Diels (TA), *Bischofia polycarpa* (H. Lév.) Airy Shaw (TB), *Pterocarya stenoptera* C. DC. (TC), and *Camphora officinarum* Nees (TD), whose old rhizomes with black tomentum are used for widely targeted metabolomics analysis. The cuticular layers of fresh rhizomes from the four groups were removed and stored in a −80 °C freezer for metabolite extraction. Three batches of rhizomes from three individual epiphytic objects, on which more than 5 specimens were grown on the one epiphytic object, were used as three biological replicates for each pattern. Methanol and acetonitrile were purchased from Merck (Darmstadt, Hesse, Germany), and formic acid was purchased from Aladdin (Shanghai, China).

### 2.2. Metabolite Extraction and Preparation of Rhizomes Samples

Metabolite extraction was conducted according to a previously published method with minor modifications [22]. The rhizome samples were freeze-dried and crushed into powder using a mixer mill (MM 400, Retsch, Haan, Germany) for 1.5 min at 30 Hz. A 0.05 g sample powder was mixed with 1.2 mL 70% methanol water (pre-cooled at 20 °C) containing internal standard components (L-2-chlorophenylalanine, purity 98%, CAS 103610-89-3) and vortexed for 30 s every 30 min a total of 6 times during the entire extraction. The extraction solution was centrifuged for 3 min at a 12,000 r·min^−1^ rotation speed, and the supernatant was filtered through a 0.22 μm microporous membrane for the UPLC-MS/MS analysis.

### 2.3. UPLC-MS/MS Measurement Based on the Widely Targeted Metabolomics Method

The methods were similar to those in the published literature [17,18]. The extraction solution was analyzed using a UPLC-ESI-MS/MS system, which comprised ultra-performance liquid chromatography (UPLC, ExionLC™ AD) and a tandem mass spectrometry system (MS/MS, Applied Biosystems QTRAP 6500, Foster City, CA, USA). The UPLC system included an Agilent SB-C18 column (1.8 μm, 2.1 mm × 100 mm) and a mobile phase (A: water; B: acetonitrile) containing 0.1% formic acid. The elution of each extraction solution was followed by a gradient program: 0–9 min, 5–95% B; 9–10 min, 95% B; 10–11.10 min, 95–5% B; 11.10–14 min, 5% B. The flow rate was 0.35 ML·min^−1^, and the injection volume was 2 μL at 40 °C column temperature.

The minimum and the maximum times were 2 and 50 ms, respectively. The targeted scan time was 1 s. The temperature of electrospray was set to 550 °C under 5500 V in the positive ion mode and −4500 V in the negative ion mode of ion spray voltage. The ion source gas I, gas II, and curtain gas were set as 50, 60, and 25 psi, respectively. The collision gas was set at 2.00 L·min^−1^. The entrance potential was 10.00 V in the positive ion mode and was −10.00 V in the negative ion mode. The collision cell exit potential was 5.00 V in the positive ion mode and was −11.00 V in the negative ion mode. The collision-induced ionization parameters were high. The QQQ scan was conducted in multiple reaction monitoring (MRM) mode at a medium level, where the MRM ion pairs were obtained by optimizing the declustering potential (DP) and collision energy (CE). A specific MRM ion pair was supervised on the basis of its components during a special period.

### 2.4. Quality Control of Extraction Samples

The mixed samples for quality control (QC) comprised those extraction solutions from the two epiphytic patterns to investigate the repeatability of the analytical samples under the same treatment conditions. The QC samples were performed after every five rhizome extractions. Repeatability was evaluated by displaying an overlap plot of the total ion chromatography (TIC).

### 2.5. Qualitative and Quantitative Analysis of Detected Metabolites

Qualitative analyses were based on an in-house database, where the targeted components were authenticated using the parent ion molecular weight (Q1), characteristic fragment ion (Q3), retention time (RT), DP, and CE values after removing isotopes and repeated signals containing K^+^, Na^+^, and NH_4_^+^ cations. Thus, the identification results were divided into three levels: level 1 included components identified based on Q3 and RT with a match score greater than 0.7; level 2 included qualitative results according to Q3 and RT with a match score between 0.5 and 0.7. Level 3 included those identified metabolites that were labeled based on Q1, Q3, RT, DP, and CE in the in-house database.

Semi-quantitative analyses of various samples were conducted using the MRM mode, which has high accuracy and repeatability without the non-targeted ions on the characteristic fragment ions. The peak areas of all metabolites were integrated, and the same metabolites from different samples were subjected to integral correction using Fraga’s methods [23]. Qualitative and quantitative mass spectrometry analyses of metabolites in the project samples were based on the MetWare database (MWDB) and multiple reaction monitoring (MRM). The mass dataset was calculated using Analyst 1.6.3 software (AB Sciex, Boston, MA, USA), and correction and integration were completed using MultiQuant 3.0 software (AB Sciex, Boston, MA, USA).

### 2.6. Multivariate Statistical Analysis and Selection of Differentially Expressed Metabolites

Chemometric analysis included principal component analysis (PCA), cluster analysis, and orthogonal partial least square discriminant analysis (OPLS-DA). PCA was performed using MetaboAnalyst 6.0 (https://www.metaboanalyst.ca/, accessed on 7 April 2024) to display the clustering and similarity using the raw dataset and illustrated with a score plot. PCA transforms the original mass dataset into several principal components (PCs) with a linear correlation relationship. The first PCs explain most of the variable profiles, and a score plot was obtained from the first two PCs. A cluster heatmap was drawn using ComplexHeatmap in R 1.01 software (www.r-project.org, accessed on 7 April 2024) using the peak area after pretreatment by unit variance scaling to compare the relative contents of the same metabolite categories in different rhizome samples. OPLS-DA was conducted using the RetaboAnalystR and OPLSR.Anal functions in R software, using a transformation dataset after Log2 and mean centering. The permutation test was used to validate the reliability of the model, which was evaluated using R2 and Q2, which reflected the explanatory percentage and prediction ability of the OPLS-DA model.

The selection of differentially expressed metabolites (DEMs) was based on the variable importance in projection (VIP) and the values of fold change (FC). Herein, metabolites with VIP > 1, FC ≥ 2, and FC ≤ 0.5 were considered DEMs between RT and TT. Furthermore, a volcanic plot was constructed by combining the log2FC and VIP values to show the upregulated and downregulated metabolites. In the following discrimination, RT was considered the control, whereas TA, TB, TC, and TD were the test groups. Therefore, a positive log2FC value indicated higher relative contents of TA, TB, TC, and TD than of RT, and vice versa. Functional annotation of the DEMs was performed using the Kyoto Encyclopedia of Genes and Genomes Database. Enrichment analysis was performed using rich factors and *p* values. A high rich factor and low *p* value indicated a strong and significant degree of enrichment. The differential abundance (DA) score was calculated on the basis of the count difference between upregulated and downregulated metabolites, reflecting the variation tendency of all metabolites in a specifical KEGG pathway.

## 3. Results

### 3.1. Metabolite Categories of Rhizomes and PCA Results between RTs and TTs of D. roosii

**Figure 1:**
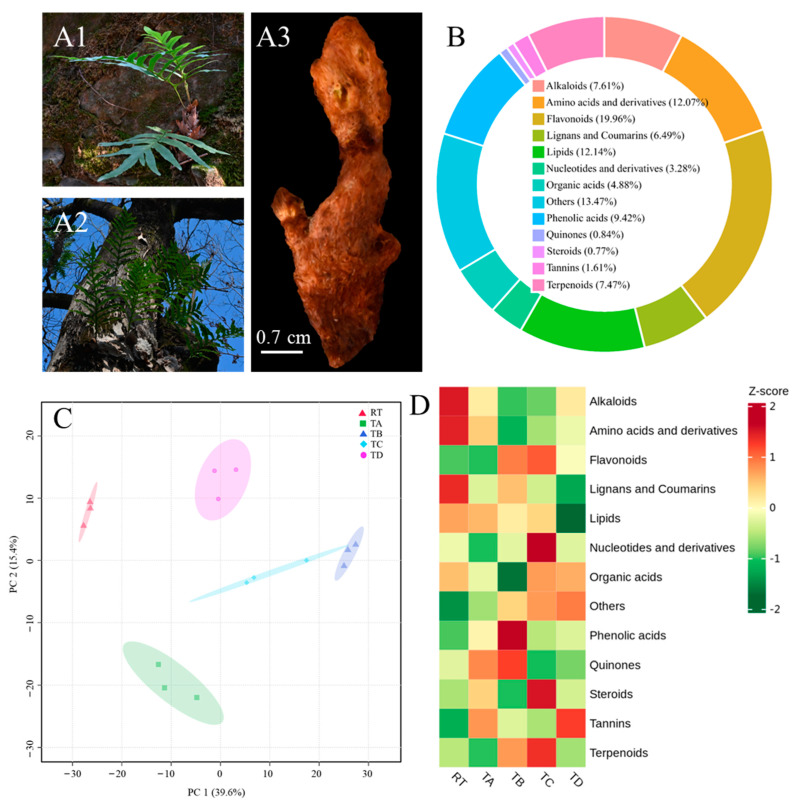
*Drynaria roosii* under two epiphytic pattern and metabolite comparison profiles. (**A1**) *D. roosii* grown on rock tunnels (RT group), where adhesion relies on moss and bits of soil in the rock tunnels. (**A2**) *D. roosii* grown on a tree trunk (four different tree species are labeled as TA, TB, TC, and TD), where rough bark provides enough adhesion surface. (**A3**) Fresh rhizome covering golden tomentum after removing fronds, and old parts are the main experimental materials. (**B**) Biochemical categories and the proportion of authenticated metabolites in these rhizomes. (**C**) Score plot of principal components analysis using all metabolite profiles based on five groups. (**D**) Heatmap of biochemical categories among various epiphytic patterns.

The wild-growth environments of *D. roosii* in the RTs and TTs are illustrated in Figure 1(A1) and Figure 1(A2), respectively. Both epiphytic patterns exhibited a humid microclimate with abundant moss surrounding the rhizomes. However, significant differences are noted in organic matter composition. The RT pattern features ample water in RTs and organic materials from the soil. In contrast, other patterns, such as TA, TC, and TD, growing on aged, rugged barks of diverse tree species, have limited soil content originating from ash or leaf debris. TB grows on the trunk surface of *Bischofia polycarpa*, the bark is not rough enough for the other three tree species, and epiphytic growth mainly depends on the amount of moss on the bark. Fresh rhizomes (Figure 1(A3)) covered with dried fronds were extracted for metabolite detection and comparison. A total of 1435 components were identified on the basis of the Q1, Q3, RT, DP, and CE values, and detailed profiles are compiled in Table S1, including parent ion molecular weight, characteristic fragment ion, molecular weight, formula, ionization model, compound name, chemical class and subclass, and CAS numbers. The overlapping TIC plots of the QC samples indicated that the RT and peak intensity of the same sample displayed strong consistency for various detection times, confirming the repeatability and reliability of our metabolite detection. Overlap plots of the total ion chromatography of QC samples of *D. roosii* are shown in Figure S1A in the positive ion mode and in Figure S1B in the negative ion mode. Ten representative MS/MS spectra of some components are displayed in Figure S1C. These metabolites were classified into 13 categories (Figure 1B), of which flavonoids, amino acids and their derivatives, and lipids were the main components, accounting for 19.96%, 12.07%, and 12.14%, respectively, of all metabolites. Additionally, alkaloids, lignans and coumarins, phenolic acids, and terpenoids exhibited substantial chemical diversity with values of more than 5%. 

PCA indicated that samples from different epiphytic patterns clustered into five groups, with the first two PCs explaining 55% of the variable information (Figure 1C). PC1 explained 39.6% of the chemical profiles and distinguished RT from TB, whereas PC2 explained 15.4% of the metabolite information and distinguished TA from RT and TD. Overall, two epiphytic patterns, RTs and TTs, were clearly clustered, and the epiphytic pattern of the TT was further divided into four subclasses owing to differences in tree species. A heatmap was used to evaluate and compare the same chemical classes in different epiphytic patterns (Figure 1D). The results showed that RT rhizomes had the highest relative content of lignans, coumarins, amino acids and their derivatives, and alkaloids. TA rhizomes exhibited the lowest relative content of all component categories. The highest relative phenolic acid and quinone contents were found in TB rhizomes. TC rhizomes had the highest relative contents of flavonoids, nucleotides and their derivatives, organic acids, steroids, and terpenoids. Tannins were only found as high-relative-content chemical components in TD rhizomes. Thus, epiphytic patterns significantly influence the content of various metabolites, even within the same epiphytic pattern in different epiphytic species.

### 3.2. Metabolite Comparison and Discrimination Results of OPLS-DA between RTs and TTs of D. roosii

**Figure 2:**
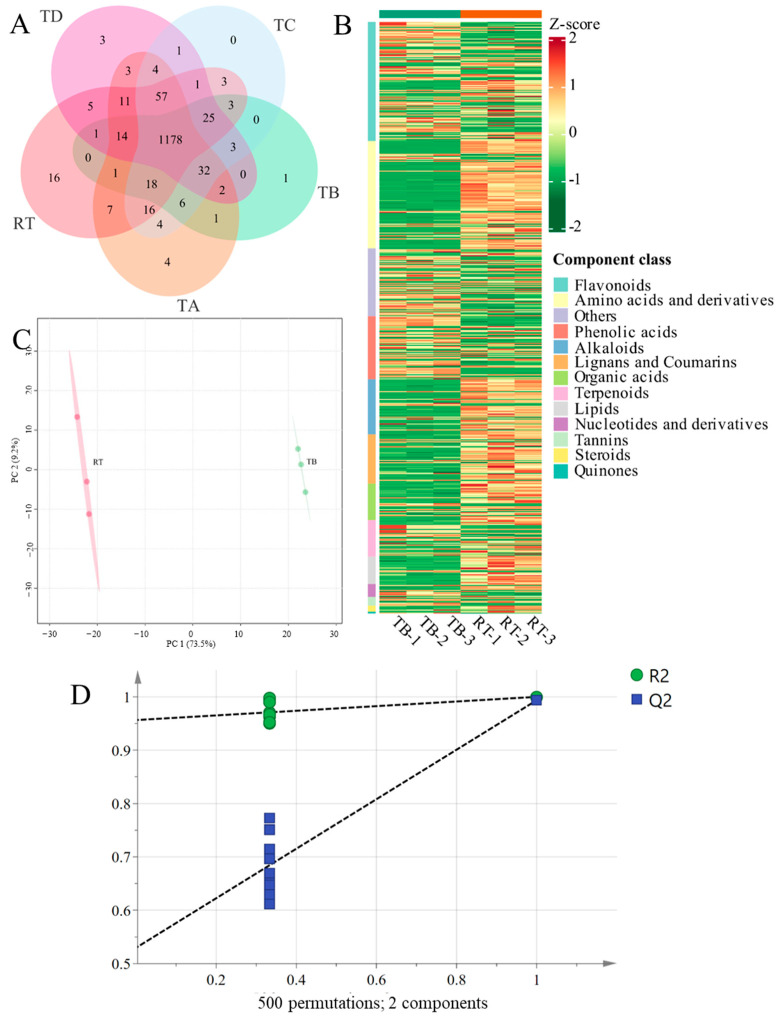
Differential metabolite analysis of *Drynaria roosii* under two epiphytic patterns. (**A**) Venn plot based on all metabolites detected in five kinds of rhizomes. (**B**) Heatmap of different biochemical categories between RT and TB rhizomes. (**C**) Score plot of orthogonal partial least square discriminate analysis using all metabolites between RT and TB. (**D**) Permutation test of partial least square discriminate analysis between RT and TB based on 500 permutation times. R2 means explanation percentages and Q2 means prediction ability.

To better identify the specific metabolites belonging to the targeted epiphytic patterns, a Venn diagram was drawn based on the 1435 metabolite profiles, with the peripheral numbers representing the special metabolites belonging to the targeted group. The counts and detailed information are presented in Figure 2A and Table 1. The results indicate that the RT pattern had a special metabolite that was not detected in the other four epiphytic species and that 16 metabolites were authenticated, including 1 alkaloid, 1 amino acid derivative, 7 flavonoids, 2 lignans, 1 lipid, 1 alcohol, 1 aldehyde, and 2 phenolic acids. The rhizomes of the TA pattern contained four special components belonging to flavonoids, lignans, and terpenoids. Cyanidin 3-*O*-sophoroside, a flavonoid component, was the only anthocyanidin detected in rhizomes with a TB pattern. No special components were detected in TC using the present method or technique. The rhizomes from the TD epiphytic pattern had three distinct compositions, two organic acids, and one phenolic acid.

In addition to the comparative analysis among the five groups, RT was regarded as the control group for the other four test groups (TA, TB, TC, and TD). Heatmap formation and OPLS-DA analysis were further conducted using all detected metabolites. The comparison group between RT and TB had the most notable clustering compared with the other three groups (TA, TC, and TD). The heatmap indicated that most of the components belonging to alkaloids, amino acids and their derivatives, lignans and coumarins, lipids, nucleotides and their derivatives, organic acids, quinones, and steroids in the RT rhizomes had higher relative contents than those in the TB rhizomes, especially amino acids and their derivatives (Figure 2B). The discrimination results indicated that TB could be clearly distinguished from RT (Figure 2C). A permutation test showed that the model had strong explanatory ability and high predictive ability. The R2 value was higher than that of Q2, reflecting that the model was robust (Figure 2D).

**Figure 3:**
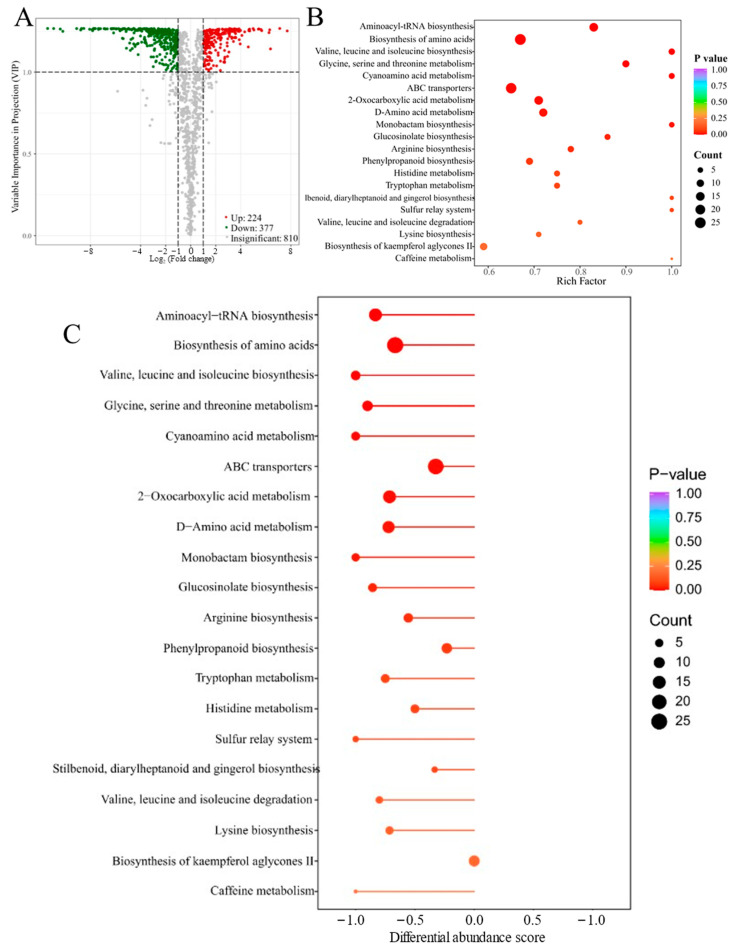
Differential metabolite analysis and enrichment results of DEMs of *Drynaria roosii* under two epiphytic patterns (RT and TB). (**A**) Volcano plot displaying the amounts of differential metabolites between RT and TB. (**B**) Enrichment pathway of differential metabolites between RT and TB. (**C**) Differential abundance score plot between RT and TB. RT is the control group; the negative differential abundance score indicated that the counts of upregulated metabolites were lower than those of downregulated metabolites between RT and TB.

DEM selection combined with VIP and FC showed that there were 601 metabolites, including 224 upregulated and 377 downregulated components, between RT and TB (Figure 3A). The detailed DEM profiles are displayed in Table S2. The top 10 most highly upregulated and downregulated differential metabolites between RT and TB of *D. roosii* are displayed in Table 2 with detailed profiles. There were five phenolic acids (homogentisic acid, 3-*O*-digalloyl quinic acid, sibiricose A3, salicin 6′-acetate, and sinapoylcaffeoyltartaric acid), two saccharides (4-*O*-galactopyranosylxylose and dihydroxyoctanoic acid glucoside), one nucleotide and its derivative (thymidine), and one tannin (sanguiin H4); glucosyl 6,9-dihydroxydec-4-enoic acid was upregulated in TB. The RT group had five amino acids and their derivatives (Val-Thr, homoarginine, γ-Glu-Tyr, *N*-α-Acetyl-l-ornithine, and methyl l-pyroglutamate), two plumeranes (5,6-dihydroxy-1H-indole-2-carboxylic acid and 3-indolepropionic acid), one flavone (vaccarin), one nucleotide (isocytosine), and one sesquiterpenoid (3-oxo-alpha-ionol 3′-(6″-malonyl)glucoside).

Additionally, 458 DEMs existed between RT and TA, comprising 234 upregulated and 224 downregulated components (Figure S2A). The detailed profiles are listed in Table S3, including the compound names and parameter values of the multivariate statistical analysis. Heatmap analysis indicated significant variation, with lignans, coumarins, alkaloids, and lipids showing many DEMs with high relative contents in RT. The comparison between RT and RC contained 479 DEMs including 174 upregulated and 305 downregulated metabolites. RT showed enrichment advantages in the relative contents of amino acids and their derivatives, alkaloids, lignans, and coumarins compared with those in TA (Figure S2A), and detailed chemical and score information is displayed in Table S4. TC had 174 upregulated DEMs and 305 downregulated DEMs compared with RT, but RT exhibited higher relative contents of most metabolites, especially amino acids, their derivatives, and lipids. The DEM profiles of RT and TD are listed in Table S5.

The top 10 DEMs between RT and TA indicated that 3 phenolic acids, 3 saccharides, 1 alkaloid, 1 amino acid, 1 other flavonoid, and 1 sesquiterpenoid were upregulated, whereas 1 amino acid, 1 lignan, 1 monoterpenoid, 1 nucleotide derivative, 2 organic acids, 1 other component, 2 phenolic acids, and 1 sesquiterpenoid were downregulated (Table S6). There were 2 amino acids and their derivatives, 1 flavanone, 1 flavonol, 1 lignan, 1 organic acid, 2 phenolic acids, and 1 saccharide that were upregulated in TC and 3 amino acids and their derivatives, 1 anthocyanidin, 1 flavone, 1 lignan, 1 monoterpenoid, 2 phenolic acids, and 1 plumerane compared with RT for the top 10 metabolites (Table S7). There was 1 amino acid and its derivative, 1 flavanone, 2 organic acids, 3 phenolic acids, 1 proanthocyanidin, and 2 saccharides upregulated in TD, whereas 1 amino acid, 1 anthocyanidin, 1 coumarin, 1 flavone, 1 flavonol, 1 lignan, 1 monoterpenoid, 2 phenolic acids, and 2 sesquiterpenoids were downregulated and included in the top 10 DEMs in TD compared to RT (Table S8).

### 3.3. Annotation and Enrichment Analysis of DEMs between RT and TT of D. roosii

Annotation of DEMs between RT and TB based on the KEGG database revealed that most DEMs mainly participated in metabolic pathways (61.84%), biosynthesis of secondary metabolites (39.47%), biosynthesis of amino acids (17.11%), and ABC transporters (15.79%); annotation percentages more than 10% and other summary annotation results are shown in Figure S5. These annotation pathways included metabolism, genetic information processing, and environmental information processing. As for the other three comparison groups, the annotation percentages varied slightly. Six pathways with > 10% DEMs were identified between the RT and TA groups. In addition to the four main annotation pathways, which were the same as the comparison group of RT&TB, the biosynthesis of cofactors and the biosynthesis of secondary metabolites also had high percentages of 12.24% and 11.22%, respectively (Figure S6). The annotation results of DEMs between RT and TC were different from the above-mentioned two comparison groups. 2-Oxocarboxylic acid metabolism and aminoacyl-tRNA biosynthesis were novel pathways with >10% DEM percentages in addition to the same four annotation pathways (Figure S7). The annotation results of DEMs between RT and TD were similar to those between RT and TA. D-animo acid metabolism was the only differential pathway with a >10% DEM percentage difference from the annotation pathways based on the DEMs between RT and TA (Figure S8).

Enrichment analysis indicated that DEMs between RT and TB mainly participated in aminoacyl-tRNA biosynthesis, biosynthesis of amino acids, ABC transporters, and D-amino acid metabolism, among others, based on the top 20 enrichment analyses (Figure 3B). Further DA score analysis indicated that those DEMs between RT and TB negatively regulated the top 20 pathways with the lowest *p* values, especially the biosynthesis of amino acids and ABC transporters. In other words, more DEMs in the RT rhizomes participated in the biosynthesis of amino acids. The enrichment analysis and DA scores were consistent with the heatmap analysis, which showed that RT had the highest relative content among the five rhizome groups. The patterns of enrichment pathways differed more with the DEMs between RT and TA than those between RT and TB; tryptophan metabolism, pyruvate metabolism, phenylalanine, tyrosine and tryptophan biosynthesis, phenylpropanoid biosynthesis, and biosynthesis of various plant secondary metabolites were the main enrichment pathways with low *p* values, high rich factors and high metabolite counts (Figure S9). The enrichment pattern between RT and TC based on the top 20 pathways showed tendencies similar to those between RT and TA, of which aminoacyl-tRNA biosynthesis, biosynthesis of amino acids, and ABC transporters were the main pathways with extremely low *p* values and high rich factors (Figure S10). The enrichment results based on the DEMs between RT and TD showed considerable variation not only in the pattern but also in the significance of D-amino acid metabolism, with monobactam biosynthesis, vitamin B6 metabolism, flavone and flavonol biosynthesis being the main enrichment pathways with low *p* values and high counts (Figure S11).

### 3.4. Differences in Chemical Composition between RT and TB of D. roosii

#### 3.4.1. Differences in Primary Metabolites between RT and TB Rhizomes

A total of 109 DEMs were identified as amino acids and their derivatives, with 95 of them upregulated in RT rhizomes. These included l-alanine, l-arginine, l-aspartic acid, l-histidine, l-leucine, l-lysine, l-proline, l-tyrosine, and l-valine with 26.13-, 1180.60-, 164.09-, 214.88-, 3.86-, 17.68-, 4.83-, 74.72-, and 7.31-fold increments, respectively. Among nucleotides and their derivatives, RT showed six downregulated and seven upregulated components, of which RT had 8.79-fold more 2′-deoxyadenosine than TB while TB had 9.49-fold more thymidine than RT. A total of 37 organic acids belonging to DEMs existed in the comparison group between RT and TB, such as 2-isopropylmalic acid, fumaric acid, hydroxypyruvic acid, malonic acid, succinic anhydride, and γ-aminobutyric acid. Of them, 81% had a higher relative content of hydroxypyruvic acid (232.05-fold). Most lipid components (26 DEMs) displayed high contents in RT, except 1 lysophosphatidyl choline (LPC) and 1 free fatty acid (hydroxypentadecenoic acid glucoside) with high relative content in TB. The DEMs of lipids included free fatty acids, glycerol esters, LPC, lysophosphatidyl ethanolamine, and sphingolipids. Saccharide comparison indicated that TB rhizomes contained 20 DEMs with high relative contents, including d-cellobiose, d-trehalose, l-xylose, raffinose, and sedoheptulose. The other five saccharides showed a tendency to be downregulated in TB compared with RT, including d-glucuronic acid, d-ribose, d-sedoheptuiose-7-phosphate, glucaric acid-1-phosphatem, and solatriose. In addition, we found two vitamin components (dehydroascorbic acid and orotic acid) downregulated in TB, and the other two vitamins (pyridoxine, pyridoxine-5′-*O*-glucoside) showed lower relative contents in RT. The relative contents of dehydroascorbic acid and orotic acid had 29.47- and 8.85-fold increments, respectively. The detailed profiles mentioned above are listed in Table S1.

#### 3.4.2. Differences in Secondary Metabolites between RT and TB

Flavonoids are the main components of the traditional Chinese medicine Drynariae Rhizoma, which is used to cure kidney deficiency, muscle tears, and loose teeth. A total of 121 flavonoids were detected and identified, including 5 anthocyanidins, 4 chalcones, 14 flavanols, 20 flavanones, 6 flavanonols, 20 flavones, 45 flavonols, 1 isoflavones, and 6 other flavonoids. The detailed flavonoid profiles are listed in Table S9. Of these, 68 flavonoids were upregulated in TB, whereas 53 components in the chemical category were downregulated. The top five flavonoids in TB were eriodictyol-7-O-(6″-acetyl)glucoside, quercetin-3-*O*-sophoroside (baimaside), dihydrocharcone-4′-O-glucoside, morin, and hesperetin-7-*O*-glucoside, with relative contents 76.10, 24.20, 17.02, 15.84, and 14.64 times higher than in RT. Eriodictyol-7-*O*-(2″-O-rhamnosyl)xyloside-4′-*O*-glucuronide, quercetin-3-O-(6″-*O*-*p*-coumaroyl)galactoside, quercetin-3-*O*-glucoside-7-*O*-rhamnoside, vitexin-2″-*O*-galactoside, and myricetin-3-*O*-glucuronide in RT had 77.35-, 49.58-, 35.72-, 29.23-, 28.12-fold higher increments than in TB.

There were 64 phenolic acids with significant differences between RT and TB, of which 44 were upregulated in TB compared with RT, such as 3-*O*-digalloyl quinic acid, sibiricose A3, homogentisic acid, picrorhizin, *p*-coumaric acid, 3-galloylshikimic acid, etc. A total of 47 upregulated alkaloids, belonging to pyridine alkaloids, pyrrole alkaloids, phenolamine, quinoline alkaloids, alkaloids, isoquinoline alkaloids, and plumerane, existed in the 56 DEMs of alkaloids with TB as the control group, such as pterolactam, retronecine, stachydrine, salicylamide, hercynine, isoquinoline, etc. A total of 50 lignans and coumarins were selected as the DEMs between RT and TB; 43 of them were downregulated in TB, such as balanophonin, balanophonin B, epipinoresinol, icariside E5, manglieside D, sesamin, symplocosin, tortoside B, epieudesmin, and vitelignin A. However, skimmin (7-hydroxycoumarin-7-*O*-glucoside) in TB was found with a 4.93-times higher content than in RT rhizomes. A total of 37 terpenoid components, including monoterpenoids, sesquiterpenoids, diterpenoids, triterpenes, and terpene, were identified as DEMs in the rhizomes of *D. roosii* between RT and TB, and 20 metabolites of these metabolites, including ebuloside, javanicolide C, procurcumenol, suavioside F, and swerosid, were downregulated in TB. In addition, we also identified two quinones (chrysophanol-1-*O*-β-d-glucoside and hydroxyAloe-emodin-8-*O*-glucoside), nine tannins (cinnamtannin A2, cinnamtannin D1, corilagin, phyllanthusiin F, proanthocyanidins, procyanidin A6, procyanidin B2, sanguiin H4, and strictinin), and six steroids (integristerone A, podecdysone C, sileneoside C, periseoside B, stigmast-4-ene-3,6-dione, and polypodin B) between RT and TB.

## 4. Discussion

*D. roosii* is the only original plant of Drynariae Rhizoma as recorded in the Chinese Pharmacopoeia, and it is widely used in medicine and as a functional food. Herbal cuisines are a traditional dietary therapy used in East and Southeast Asian countries. Chinese people in the Guangdong and Fujian provinces commonly consume soup made from fresh or dried rhizomes with chicken or pig bones, owing to their potential function in body health brought about by the abundant primary metabolites and secondary components. The present research mainly concentrated on secondary metabolites, especially flavonoid components, which are considered the main active components for bone and muscle breakage [1,24,25]. The widely targeted metabolomic analysis revealed the diversity of primary and secondary metabolites in herbal medicines, and the main differential metabolites category provided more comprehensive references for deep utilization, rather than depending on minor index components. We compared the metabolites of *D. roosii* rhizomes from different epiphytic patterns to reveal variation in the DEMs.

### 4.1. Epiphytic Patterns between RT and TT Generated Impacts on Metabolite Accumulation of D. roosii

The comparison between RT and TT indicated that RT had high relative contents of four types of metabolites, including alkaloids, amino acids and their derivatives, lignans and coumarins, and lipids. The metabolites of epiphytic plants are susceptible to epiphytic patterns because of differences in soil properties such as soil total phosphorus, pH, and the microbiome [26,27,28]. Published studies have revealed that the naringin content in the rhizomes of *D. roosii* on cliffs was significantly higher than that in TTs, when collected from Hunan Province, China [29]. Our study detected marker components for quality control, but the differences were not significant. However, we found that the TT modes, especially TB, TC, and TD, accumulated flavonoid components, and the total flavonoids were validated as the main active ingredients for the proliferation and osteogenic differentiation of rat dental pulp stem cells [2], as well as against acute renal failure [5]. The results could be interpreted to mean that the collected samples of the two studies were from two provinces of China and that the microclimate could generate potential influences on the metabolites of rhizomes, even in the same epiphytic pattern.

Amino acids and their derivatives displayed higher relative contents in RT than in TT. These primary metabolites are gaining interest in plant immunity’s role against pathogenesis and resistance [30,31]. We found that l-alanine, l-arginine, l-aspartic acid, l-histidine, l-leucine, l-lysine, l-proline, l-tyrosine, and l-valine had higher contents in RT rhizomes, of which l-leucine, l-lysine, l-tyrosine, and l-valine were reported to have an increased tendency of occurring in pathogenesis [32]. The difference in amino acids was consistent with the field investigation that *D. roosii* grown in rock tunnels had harsh soil conditions, and little substance could support their development of rhizomes and fronds.

Alkaloids are natural antibiotics with a wide antibacterial spectrum and low tendency to induce drug resistance; these properties enable widespread selection for new antibacterial drugs [33]. Current information indicates that naturally occurring alkaloids showed antiproliferative and anticancer effects both in vitro and in vivo, with enormous potential for new drug development for cancer therapy and management [34,35]. The difference between RT and TT in terms of the type and concentration of alkaloids was evident, which led to clinical differences when the two rhizome types were utilized. Prerolactam, a differentially expressed alkaloid, can be designed to improve antifungal activity [36]. 

Skimmin was extracted and validated as an effective agent for slowing the progression of membranous glomerulonephritis [37] and suppressing streptozotocin-induced diabetic nephropathy [38]. These effects were similar to the traditional efficacy of crude herbs against kidney deficiency. The relative content of the component in TB was found to be 4.93 times as high as that in RT rhizomes, which might cause potential differences in clinical usage. In addition, we identified some tannin components, such as corilagin, sanguiin H4, and strictinin, which have been confirmed to have anti-tumor effects, anti-inflammatory effects, hepatoprotective activity, and antiviral effects [39,40,41].

### 4.2. Primary and Secondary Metabolites Displayed Differences among Different Tree Species in the Epiphytic Patterns of Tree Trunks of D. roosii

We believe that *D. roosii* could select some special ligneous plants that are suitable for spore germination. Most epiphytic trees have a long growth period with rough bark and moss on the surface. Our heatmap analysis indicated that the four epiphytic trees displayed special advantages in the accumulation of different metabolites. The three epiphytic trees of TB showed the highest relative content of phenolic acids and quinones, whereas tannins displayed the highest enrichment in TD. The metabolites enriched in the TC group were flavonoids, nucleotides and their derivatives, steroids, and terpenoids. Flavonoids and terpenoids are the main chemical components used in fracture treatment, bone injury, and kidney protection [42,43]. Thus, the pharmaceutical effects of Drynariae Rhizoma from various epiphytic trunks may differ because of differences in the types and concentrations of metabolites. Substance selection should be focused on farming households. The selection of tree species was also a focus when foresters conducted compound planting between trees and *D. roosii*.

### 4.3. The Selection of Cultivation Patterns Should Pay Attention to Further Large-Scale Production

The cultivation of fern species is more difficult than that of most medicinal plants because of their strict requirements for their growth environment and their special plant structure [44,45]. The entire growth period of ferns requires two vital stages, the prothallium and sporophore stages, in which germination and development are sensitive to cultivation patterns [46]. Ferns cannot increase water-use efficiency under drought conditions to increase fitness pressure due to drought because of the evolution of stomatal closure compared with seed plants [47]. Therefore, the suitability of cultivation patterns is important to ensure healthy growth for the germination of spores and frond development as well as the formation of next-generation spores. Cultivation patterns have a considerable effect on the growth and development of rhizomes and even spore breeding. Substrate type and size determine the germination rate of spores and the growth vigor of the prothallium during spore breeding [48], and the difference in substrates affects the survival rates during cutting propagation [49]. This further alters the metabolites synthesized in the rhizomes of *D. roosii*. However, the yield and contents of metabolites should be considered because RT often leads to low yield, according to field investigations, and because rhizomes in TT modes have a high flavonoid content. Thus, selection and blending have considerable potential for further studies.

### 4.4. Metabolite Analysis Is Not the Only Research Target in Terms of the Effect Caused by the Differences in Epiphytic Patterns

Epiphytes play a vital role in driving the global diversity gradient, and wild resources should be protected [50]. *D. roosii*, a traditional herb, has been destroyed owing to excessive collection for commercial utilization, leading to an ecological imbalance. Therefore, artificial cultivation is necessary to protect wild resources. Scientific research on the selection of growth substrates is the first step in the large-scale cultivation of epiphytic plants. The present study concluded that epiphytic patterns cause variations in the primary and secondary metabolites. We speculated that the formation of DEMs is directly affected by substrates with different epiphytic patterns, which have different soil properties and microorganisms. Similar research on *D. catenatum*, an epiphytic species found on trees or cliffs, has shown that microbial communities have a substantial effect on chemical components and stem polysaccharides because of bacterial and fungal variations in abundance, diversity, and community structure in various cultivation modes [27].

In addition to the investigation of DEMs between RT and TT, growth years and tissues also affected the metabolites. Naringin mainly accumulated in the rhizomes, and new rhizomes displayed enrichment advantages, whereas neoeriocitrtin accumulated and increased gradually over time [13,14]. The metabolites of other categories and influencing factors with variations in rhizome expansion and growth years must be investigated.

## 5. Conclusions

Rhizomes of *D. roosii* collected from the two epiphytic patterns, denoted as RT and TT, were subjected to extraction to discern differences in metabolites using a widely targeted metabolomics approach. PCA effectively distinguished the distinct epiphytic patterns into five clusters, one for RT and four for TT, each corresponding to different tree trunk species. Metabolite block analysis highlighted the enrichment categories in the RT pattern, including alkaloids, amino acids and their derivatives, lignans, coumarins, and lipids. Conversely, nine other metabolite categories exhibited elevated relative contents in various TT patterns. The variances in primary and secondary metabolites between RT and TT may lead to distinct efficacies in the development of tonic diets and patent medicines. A comparative diversity assessment between RT and TT offers valuable insights into selecting cultivation substrates and grading collective rhizomes in natural habitats of *D. roosii*.

## Figures and Tables

**Table 1:** Special biochemical components of each group of *Drynaria roosii*.

Epiphytic Patterns	Biochemical Components	Categories
Rock tunnels	*N*-Acetyl-5-hydroxytryptamine	Alkaloids
	4-amino-5-oxo-5-(pentylamino)pentanoic acid	Amino acids and derivatives
	Cyanidin-3,5-*O*-diglucoside (Cyanin)	Flavonoids
	Gossypetin-3-*O*-rutinoside	Flavonoids
	Kaempferol-3,7-*O*-dirhamnoside (Kaempferitrin)	Flavonoids
	Kaempferol-3-(2″-acetylrhamnoside)	Flavonoids
	Kaempferol-3-*O*-(2″-*O*-acetyl)glucoside	Flavonoids
	Kuraridin	Flavonoids
	Myricetin-3-*O*-glucuronide	Flavonoids
	Epipinoresinol	Lignans
	Pinoresinol	Lignans
	Dodecanedioic aicd	Lipids
	Demethyl coniferin	Others—alcohol
	4-Hydroxy-3,5-Dimethoxybenzaldehyde (Syringaldehyde)	Others—aldehyde
	Isopropyl ferulate	Phenolic acids
	*p*-Coumaryl alcohol	Phenolic acids
Tree A	Cyanidin-3-O-(6″-*O*-acetyl)glucoside-5-*O*-glucoside	Flavonoids
	Lyoniresinol-9′-*O*-xyloside (Lyoniside)	Lignans
	Pterosin O	Terpenoids
	Sarcaglaboside A	Terpenoids
Tree B	Cyanidin 3-*O*-sophoroside	Flavonoids
Tree C	None	-
Tree D	1′-*O*-Galactoyl *p*-Coumaric acid	Phenolic acids
	2,4-Hexadienoic acid	Organic acids
	2-Methyl-4-pentenoic Acid	Organic acids

**Table 2:** Top 10 most highly upregulated and downregulated differential metabolites between RT and TB of *Drynaria roosii*.

Metabolites	Category	VIP Value	*p* Value	Log2(FC)
Thymidine	Nucleotides and derivatives	1.2637	0.0002	3.246
Glucosyl 6,9-dihydroxydec-4-enoic acid	Others	1.2659	0.0039	4.983
Homogentisic acid	Phenolic acids	1.2648	0.0046	3.493
3-*O*-Digalloyl quinic acid	Phenolic acids	1.2638	0.0168	5.444
Sibiricose A3	Phenolic acids	1.2625	0.0171	4.700
Salicin 6′-Acetate	Phenolic acids	1.2623	0.0121	3.649
Sinapoylcaffeoyltartaric acid	Phenolic acids	1.2616	0.0136	3.567
4-*O*-galactopyranosylxylose	Saccharides	1.2665	0.0003	3.105
Dihydroxyoctanoic acid glucoside	Saccharides	1.2624	0.0193	5.404
Sanguiin H4	Tannin	1.2628	0.0005	2.268
Val-Thr	Amino acids and derivatives	1.2665	0.0028	−11.422
Homoarginine	Amino acids and derivatives	1.2665	0.0013	−8.528
γ-Glu-Tyr	Amino acids and derivatives	1.2663	0.0027	−6.123
*N*-α-Acetyl-l-ornithine	Amino acids and derivatives	1.2662	0.0082	−10.937
Methyl l-pyroglutamate	Amino acids and derivatives	1.2660	0.0030	−5.311
Vaccarin	Flavones	1.2665	0.0005	−4.562
Isocytosine	Nucleotides and derivatives	1.2665	0.0013	−8.715
5,6-Dihydroxy-1H-indole-2-carboxylic acid	Plumerane	1.2662	0.0011	−4.038
3-Indolepropionic acid	Plumerane	1.2659	0.0083	−7.365
3-Oxo-Alpha-Ionol 3′-(6″-Malonyl)Glucoside	Sesquiterpenoids	1.2659	0.0033	−4.972

## Data Availability

Data will be available from the authors upon reasonable request due to privacy or ethical restrictions.

## Supplementary Materials

The following supporting information can be downloaded at https://www.mdpi.com/article/10.3390/metabo14080409/s1: Figure S1. Overlap plots of total ion chromatography of quality control samples of *Drynaria roosii*; Figure S2. Differential metabolite analysis of *Drynaria roosii* under two epiphytic patterns; Figure S3. Differential metabolite analysis of *Drynaria roosii* under two epiphytic patterns; Figure S4. Differential metabolite analysis of *Drynaria roosii* under two epiphytic patterns; Figure S5. The annotation results of DEMs of *Drynaria roosii* between RT and TB based on the KEGG database; Figure S6. The annotation results of DEMs of *Drynaria roosii* between RT and TA based on the KEGG database; Figure S7. The annotation results of DEMs of *Drynaria roosii* between RT and TC based on the KEGG database; Figure S8. The annotation results of DEMs of *Drynaria roosii* between RT and TD based on the KEGG database; Figure S9. Top 20 enrichment pathways of differential metabolites between RT and TA; Figure S10. Top 20 enrichment pathways of differential metabolites between RT and TC; Figure S11. Top 20 enrichment pathways of differential metabolites between RT and TD; Table S1. The detailed metabolite information detected in rhizomes of *Drynaria roosii*; Table S2. The different metabolite information detected in rhizomes of *Drynaria roosii* between RT and TB; Table S3. The different metabolite information detected in rhizomes of *Drynaria roosii* between RT and TA; Table S4. The different metabolite information detected in rhizomes of *Drynaria roosii* between RT and TC; Table S5. The different metabolite information detected in rhizomes of *Drynaria roosii* between RT and TD; Table S6. Top 10 most highly upregulated and downregulated differential metabolites between RT and TA of *Drynaria roosii*; Table S7. Top 10 most highly upregulated and downregulated differential metabolites between RT and TC of *Drynaria roosii*; Table S8. Top 10 most highly upregulated and downregulated differential metabolites between RT and TD of *Drynaria roosii*; Table S9. Detailed flavonoids profiles between RT and TB of *Drynaria roosii*.
