# Supplementary material for: Epiphytic Patterns Impacting Metabolite Diversity of Drynaria roosii Rhizomes Based on Widely Targeted Metabolomics
Source: Metabolites. 2024 Jul 26;14(8):409. doi: 10.3390/metabo14080409 (PMC11356174; doi:10.3390/metabo14080409)

Article

Epiphytic Patterns Impacting Metabolite Diversity of *Drynaria roosii* Rhizomes Based on Widely Targeted Metabolomics

Nana Chang [1,2], Xianping Yang [3], Xiaoqing Wang [4], Chao Chen [4], Chu Wang [1,2], Yang Xu [1,2], Hengyu Huang [5,*] and Ye Wang [1,2,*]

[1] Jiangxi Province Key Laboratory of Sustainable Utilization of Traditional Chinese Medicine Resources, Institute of Traditional Chinese Medicine Health Industry, China Academy of Chinese Medical Sciences, Nanchang 330115, China

[2] Jiangxi Institute of Traditional Chinese Medicine Health Industry, Nanchang 330115, China

[3] Dexing Research and Training Center, Dexing Academy of Traditional Chinese Medicine, Dexing 334213, China

[4] Jiangxi Provincial Institute of Traditional Chinese Medicine, Nanchang 330046, China

[5] College of Traditional Chinese Medicine, Yunnan University of Chinese Medicine, Kunming 650500, China

* Correspondence: hhyhhy96@163.com (H.H.); wwyy910@126.com (Y.W.); Tel.: +86-0871-6503-3564 (H.H.); +86-0791-8306-9969 (Y.W.)

Figure S1. Overlap plots of total ion chromatography of quality control samples of *Drynaria roosii*. (A) Positive ion mode. (B) Negative ion mode. (C) MS/MS spectra of 10 representative components.

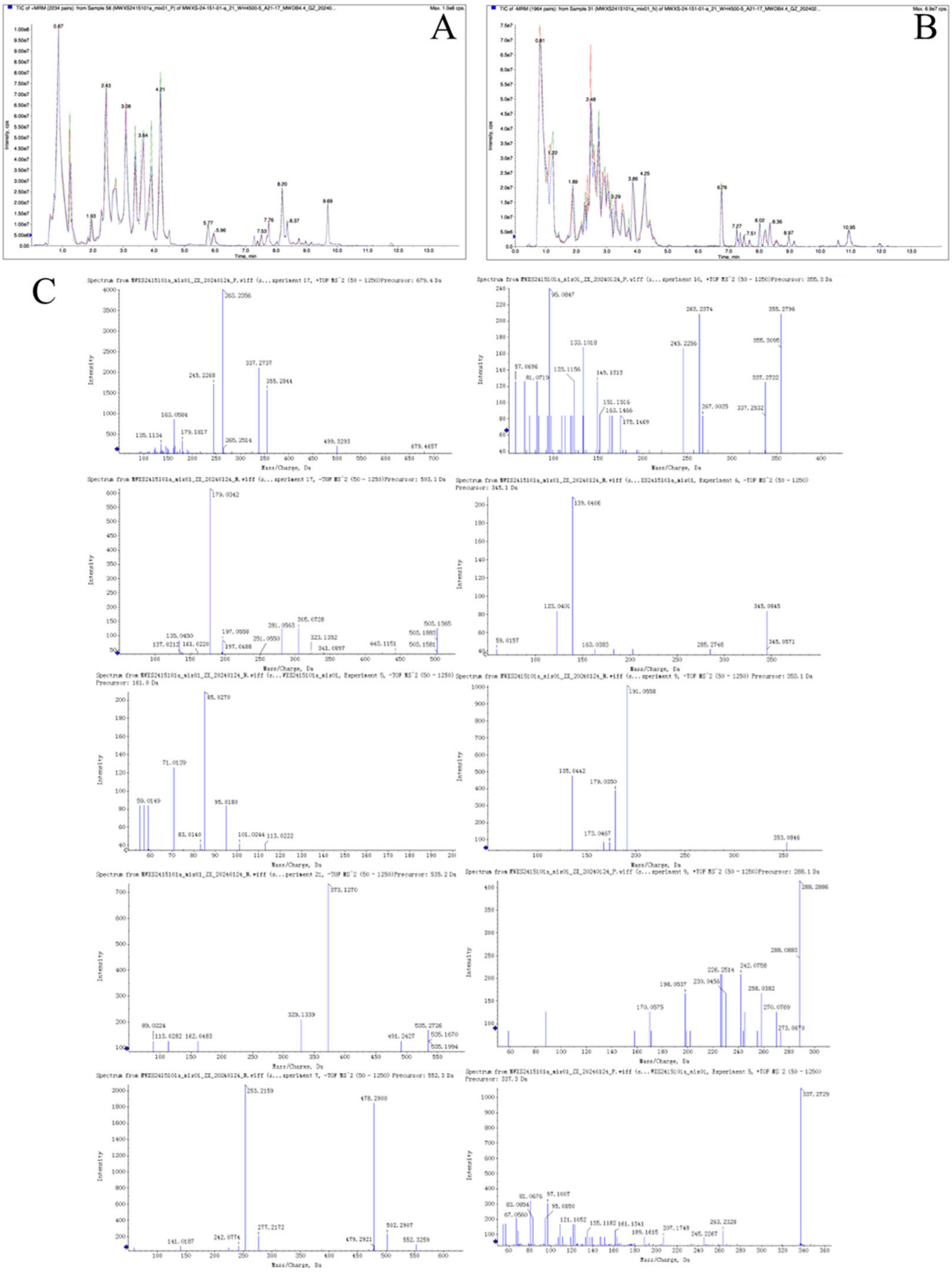

Figure S2. Differential metabolite analysis of *Drynaria roosii* under two epiphytic patterns. (A) Volcano plot displaying the amounts of differential metabolites between RT and TA. (B) Heatmap of different biochemical categories between RT and TA in the rhizome.

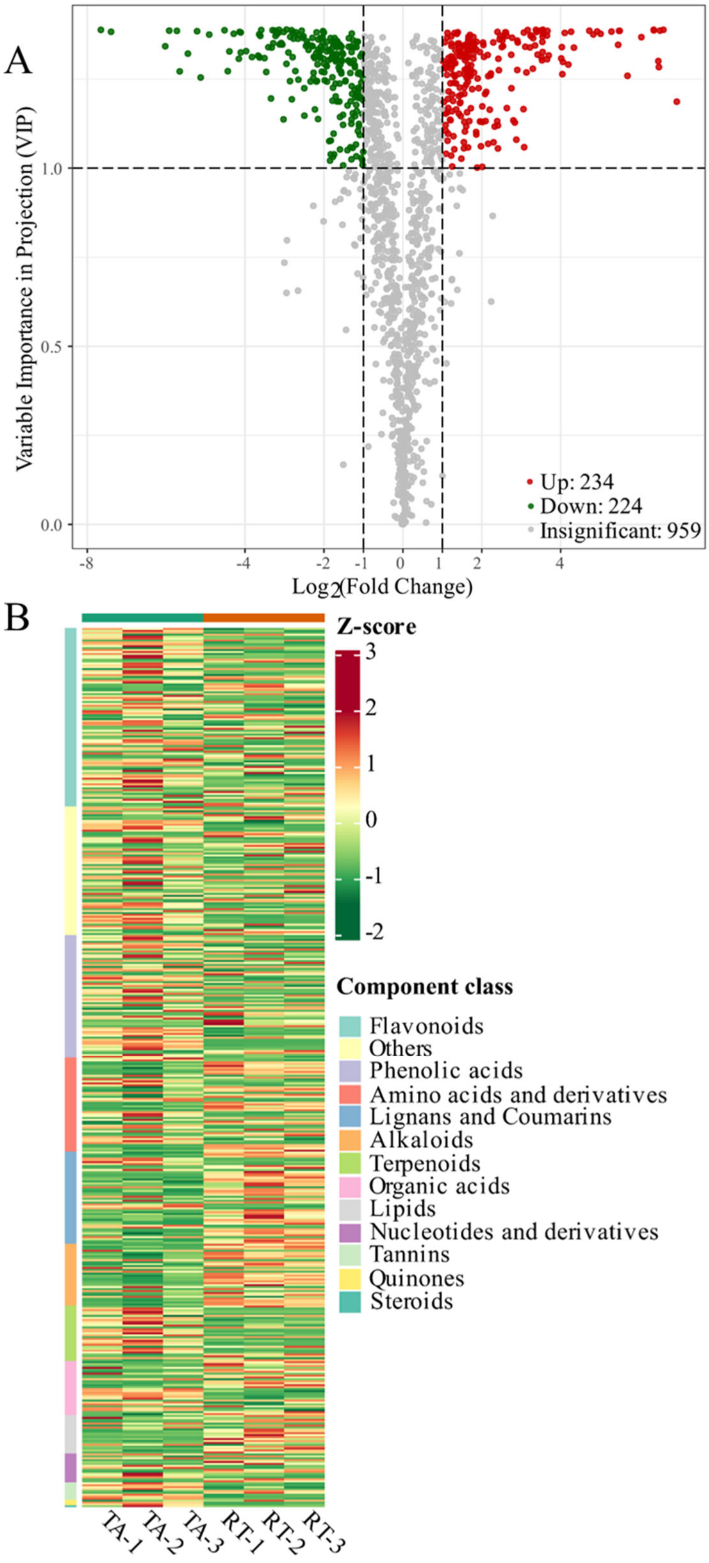

Figure S3. Differential metabolite analysis of *Drynaria roosii* under two epiphytic patterns. (A) Volcano plot displaying the amounts of differential metabolites between RT and TC. (B) Heatmap of different biochemical categories between RT and TC in the rhizome.

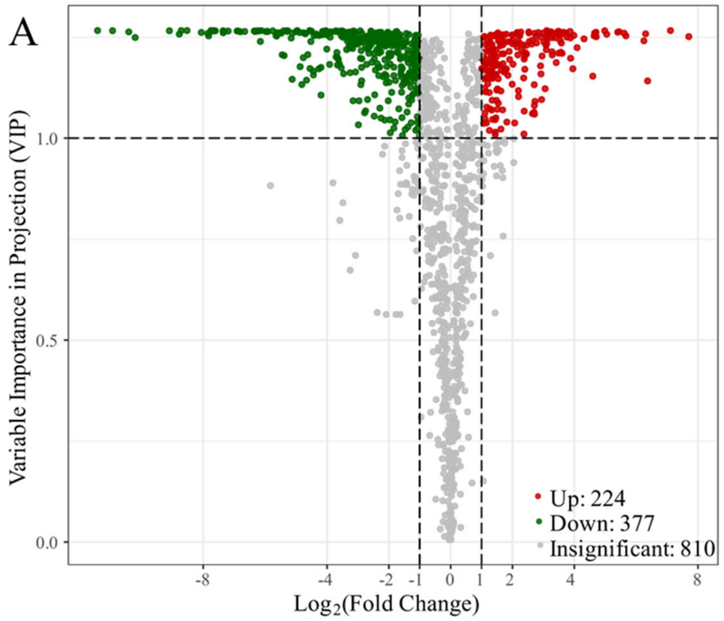

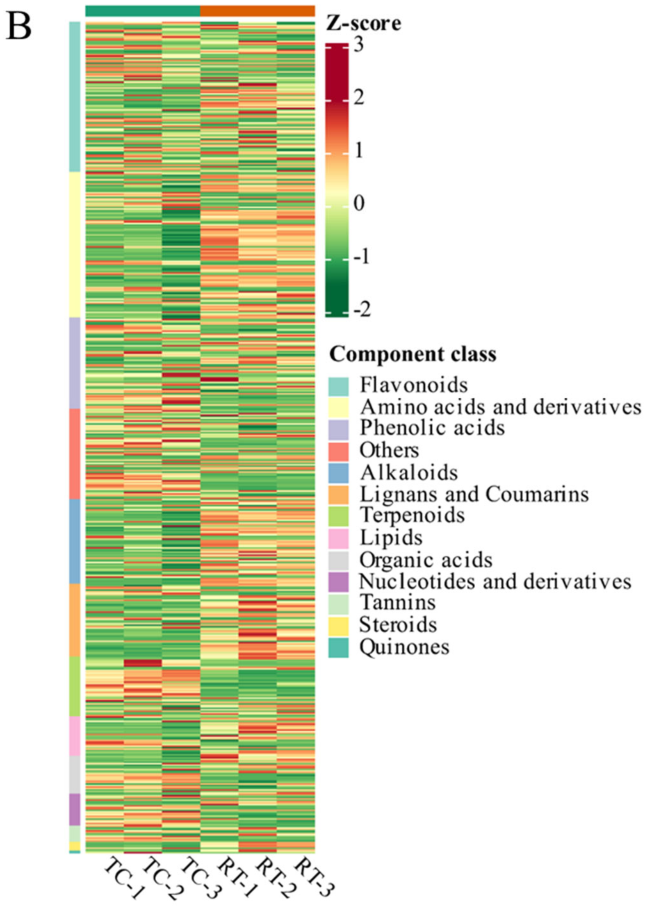

Figure S4. Differential metabolite analysis of *Drynaria roosii* under two epiphytic patterns. (A) Volcano plot displaying the amounts of differential metabolites between RT and TC. (B) Heatmap of different biochemical categories between RT and TC in the rhizome.

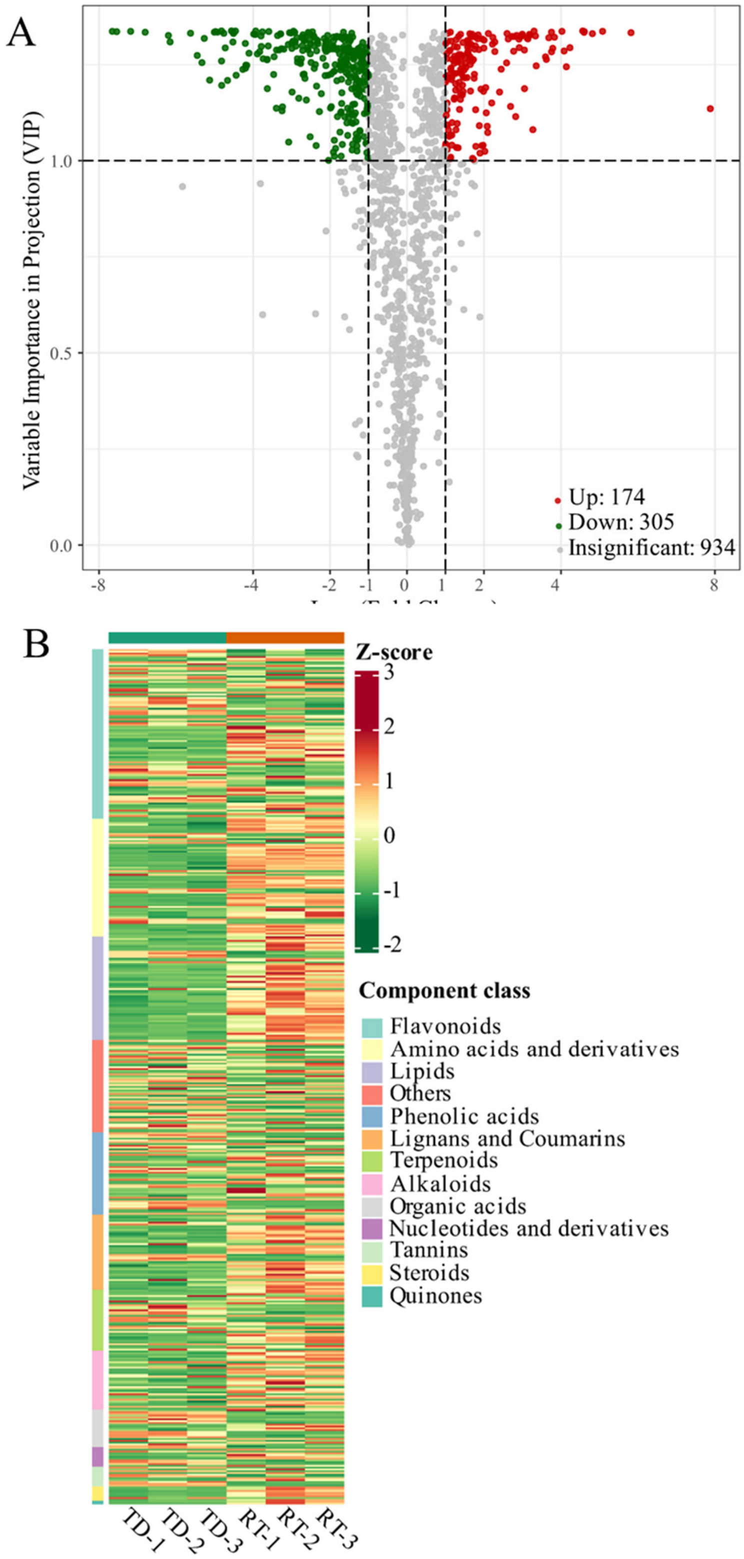

Figure S5. The annotation results of DEMs of *Drynaria roosii* between RT and TB based on the KEGG database.

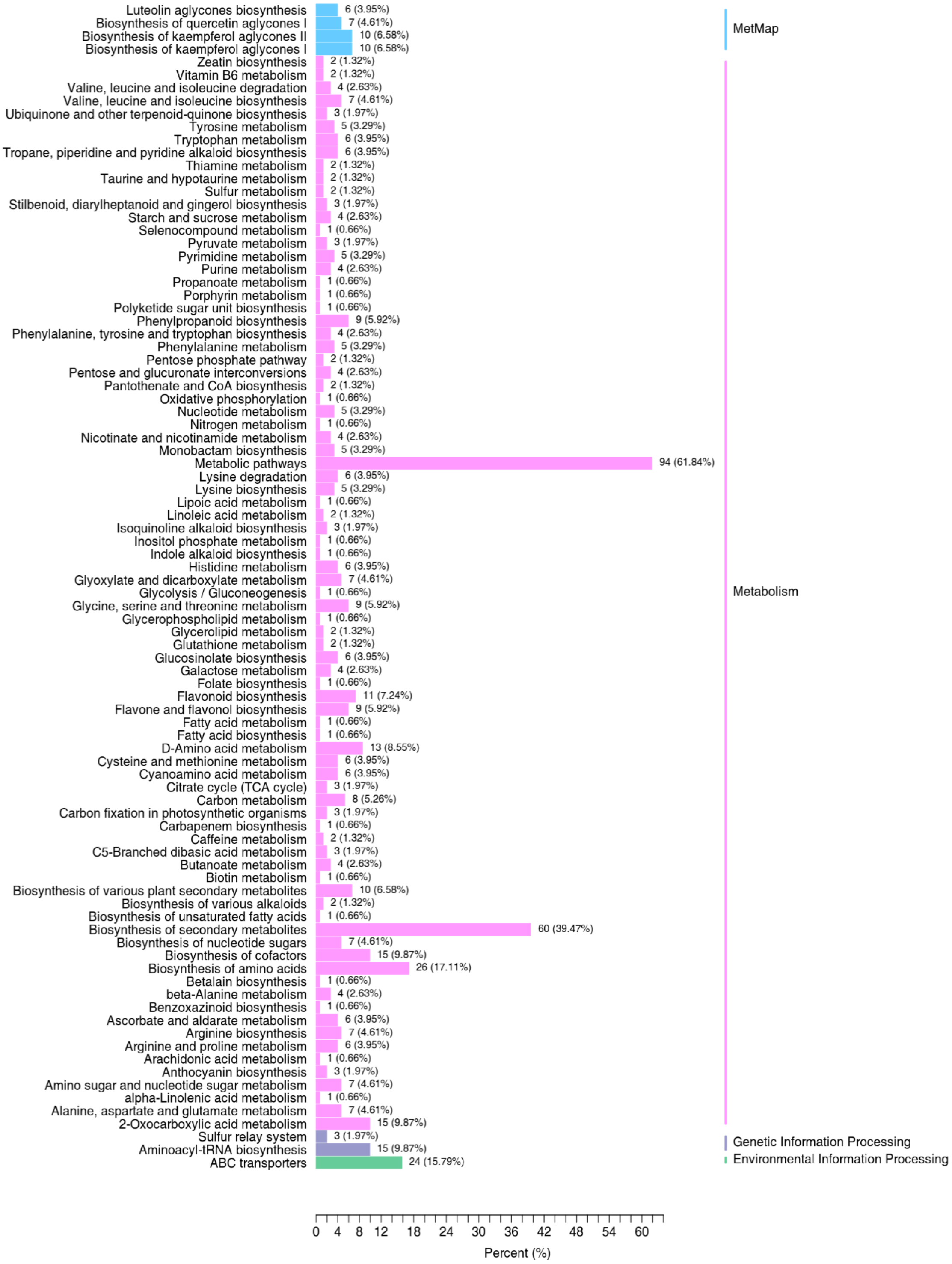

Figure S6. The annotation results of DEMs of *Drynaria roosii* between RT and TA based on the KEGG database.

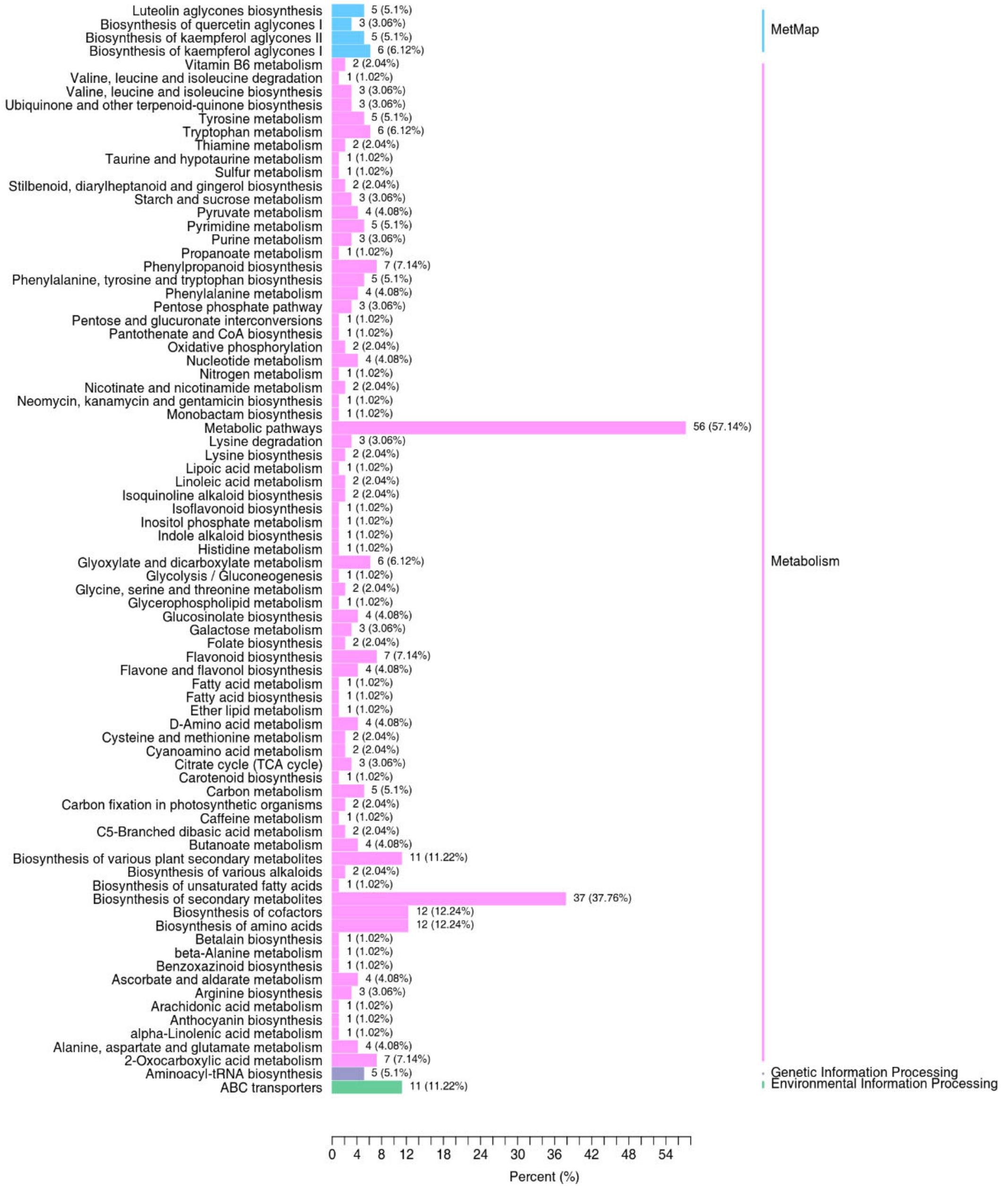

Figure S7. The annotation results of DEMs of *Drynaria roosii* between RT and TC based on the KEGG database.

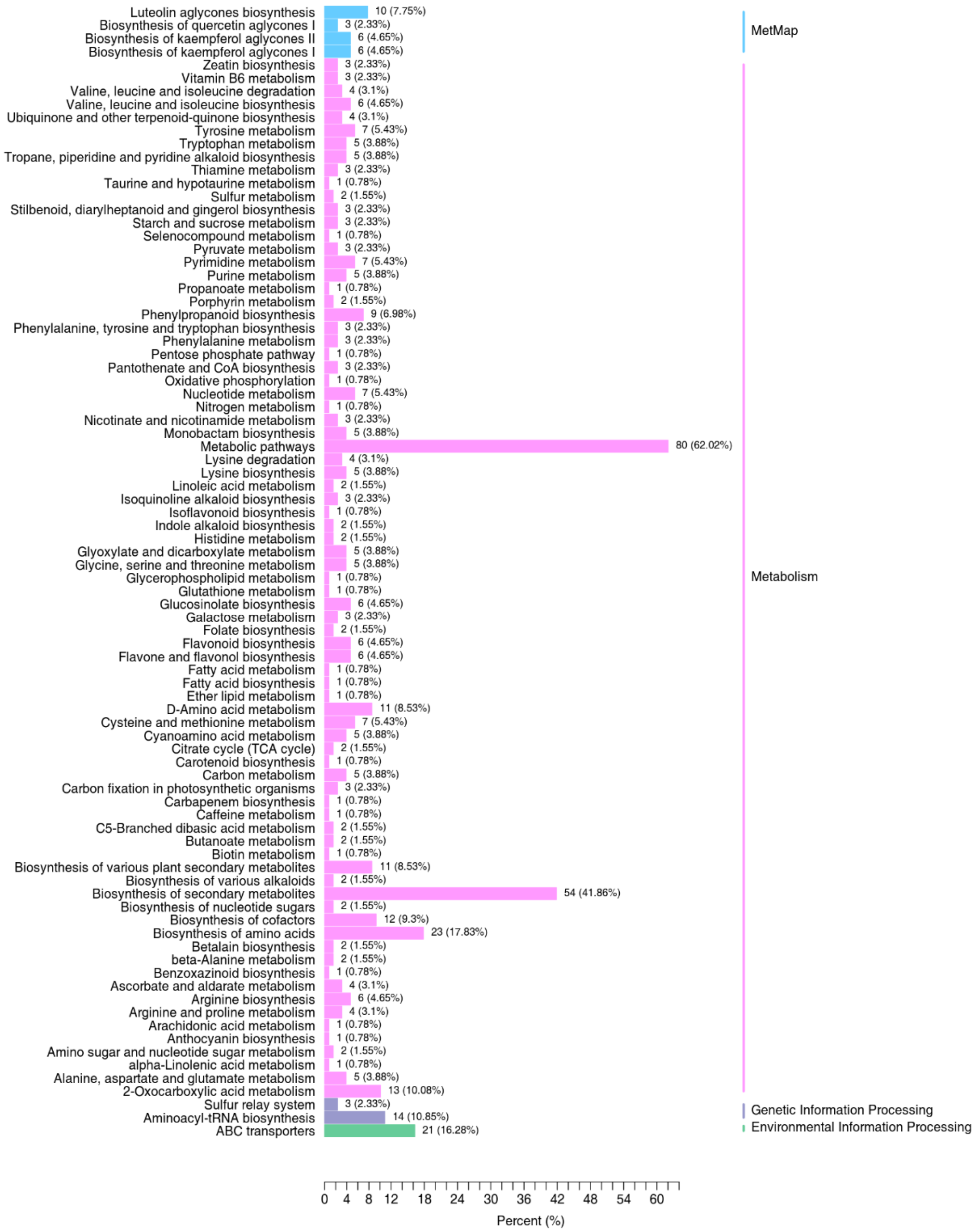

Figure S8. The annotation results of DEMs of *Drynaria roosii* between RT and TD based on the KEGG database.

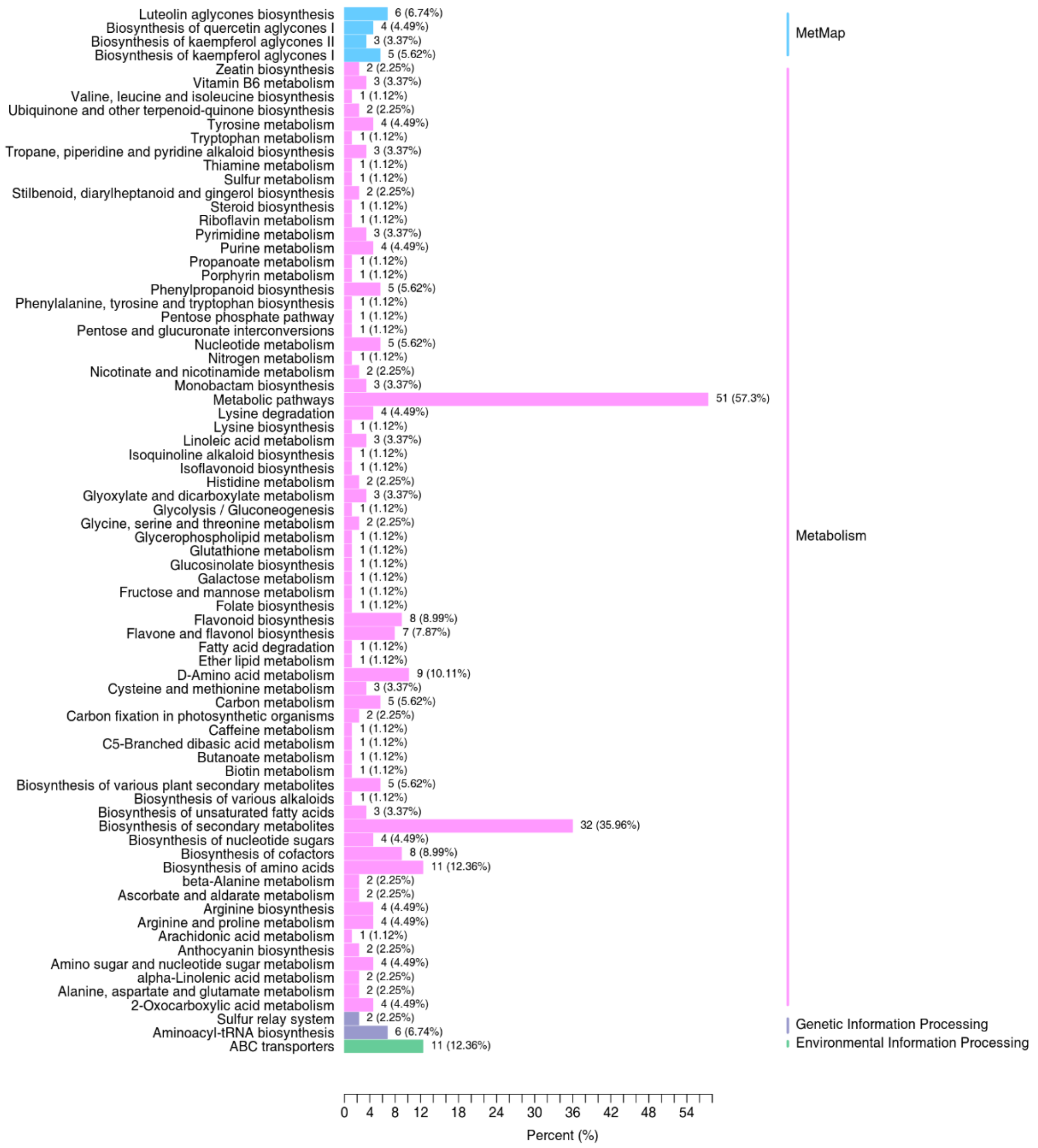

Figure S9. Top 20 enrichment pathways of differential metabolites between RT and TA.

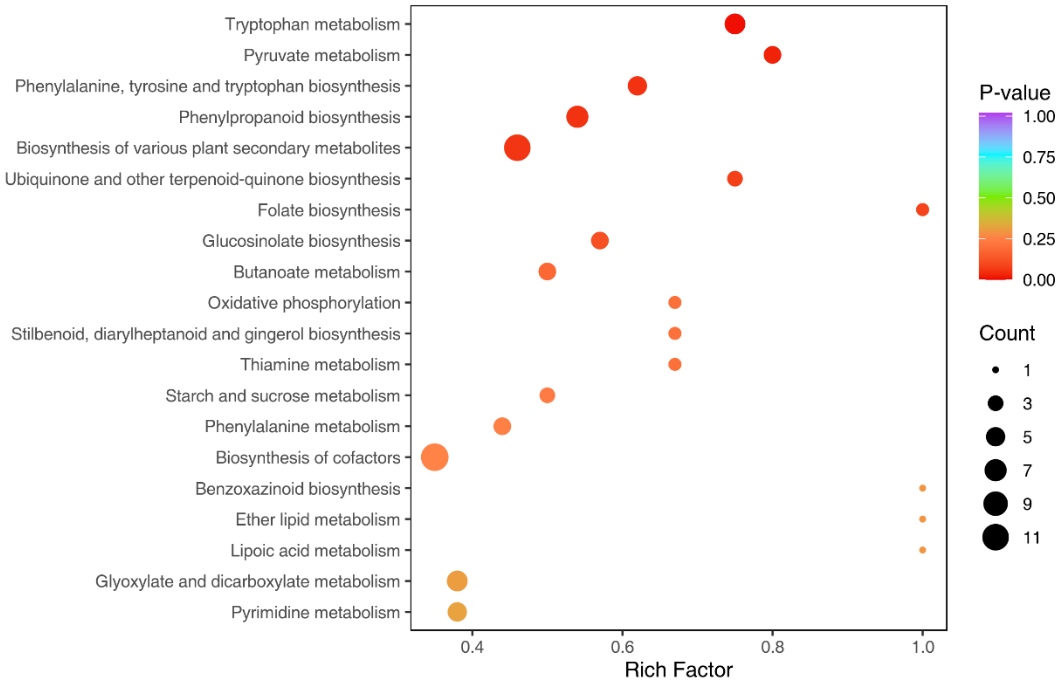

Figure S10. Top 20 enrichment pathways of differential metabolites between RT and TC.

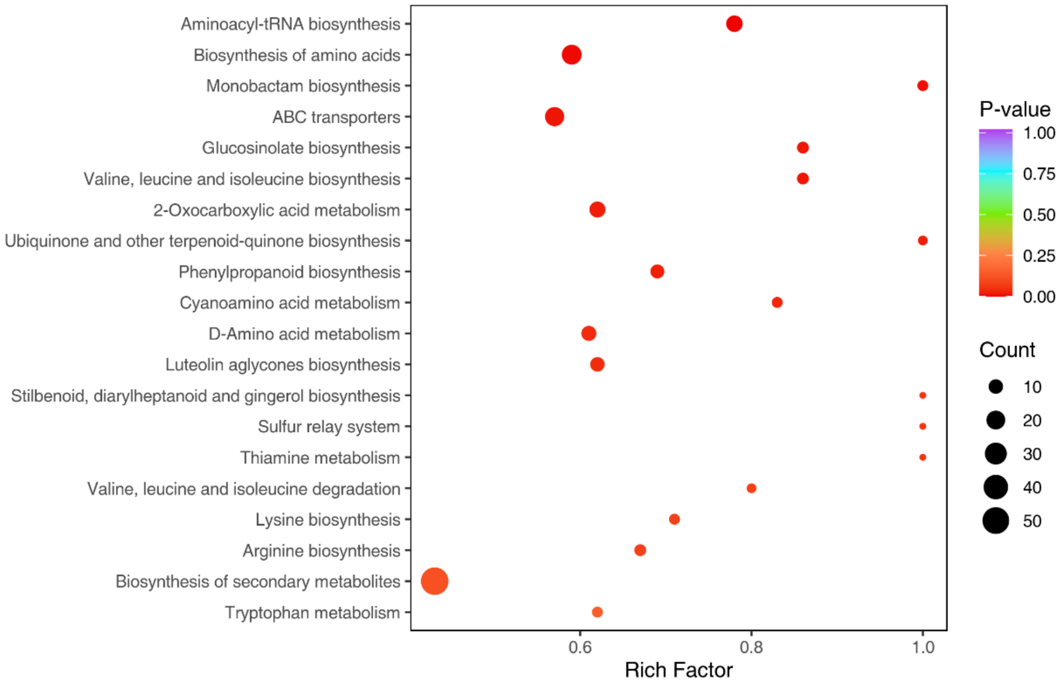

Figure S11. Top 20 enrichment pathways of differential metabolites between RT and TD.

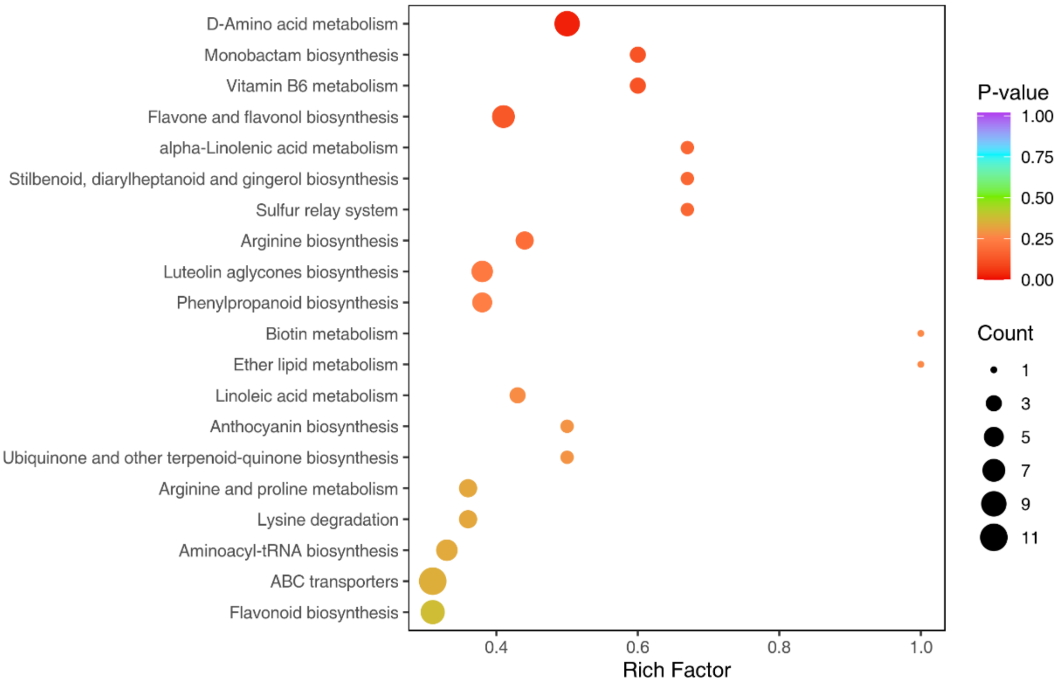

Supplement: Supplementary file 1 [file metabolites-14-00409-s001.zip › Supplementary figures.pdf]
